# Designing Advanced Soft Magnetic Powder Cores with Ultralow Energy Loss by Combining Ultrasonic and Static Compaction

**DOI:** 10.1002/advs.202522847

**Published:** 2026-01-26

**Authors:** Xiaoying Huang, Dongming Zhu, Xiao Jin, Yanan Chen, Meng Gao, Xuanyuan Zhang, Min Nie, Bingnan Yao, Wei Xu, Lijian Song, Juntao Huo, Wenbo Wang, Xiaojun Zhao, Mingliang Xiang, Jun‐Qiang Wang, Yan Zhang

**Affiliations:** ^1^ Ningbo Institute of Materials Technology and Engineering Chinese Academy of Science Zhejiang Key Laboratory of Magnetic Materials and Applications Ningbo China; ^2^ School of Materials Science and Chemical Engineering Ningbo University Ningbo China; ^3^ Center of Materials Science and Optoelectronics Engineering University of Chinese Academy of Sciences Beijing China; ^4^ Department of Electrical Engineering North China Electric Power University Baoding China; ^5^ Shenzhen Sunlord Electronics Co., Ltd Shenzhen China; ^6^ North China Electric Power University Beijing China

**Keywords:** amorphous, high frequency, powder core, powder pressing, ultrasonic vibration pressing‐cold pressing (UVP‐CP)

## Abstract

Power inductors are critical in electronics and automotive systems, with integrated inductors gaining attention for miniaturization and high power efficiency. Iron‐based amorphous/nanocrystalline soft magnetic powders offer high permeability and low core loss, yet their high strength and brittleness pose compaction challenges. This study introduces an ultrasonic vibration‐assisted cold pressing (UVP‐CP) technique, which promotes particle rearrangement and interlocking by reducing interparticle friction, enabling the fabrication of dense powder cores under reduced forming pressure. Compared with conventionally cold pressed cores fabricated from the same powder system, the UVP‐CP samples exhibit improved soft magnetic performance, achieving an effective permeability (*µ*_e_) of 47.8 at 10 MHz and a core loss of 175.8 mW/cm^3^ at 50 mT/100 kHz. These values represent a 21.0% improvement in permeability and a 43.4% reduction in core loss over conventional counterparts, establishing a new paradigm for high performance integrated inductor fabrication.

## Graphical abstract

Increasing the density and homogeneity of soft magnetic composites are critical for achieving high power density and low core loss. This work proposes a novel strategy by combining ultrasonic‐assisted pressing and cold pressing. In this line, the density and permeability of magnetic core have been increased a lot, while the core loss is decreased substantially.

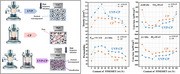

## 1 Introduction

Amidst the global transition toward sustainable energy paradigms and accelerating societal electrification, high‐efficiency inductive components have emerged as critical enablers for advanced electrical energy management, storage, and distribution systems [1, 2, 3]. In addition, with the rapid development of wide bandgap semiconductors, power conversion devices are required to operate at higher frequencies [4]. Advanced inductive systems employing powder cores are required to simultaneously deliver high power density, high frequency stability, and low loss while maintaining geometric design flexibility [5, 6, 7]. However, given the rapid development of the magnetic component industry, practical applications have imposed increasingly stringent demands on the manufacturing capabilities of powder cores.

The preparation process of powder core involves magnetic powder preparation, insulation coating, compression molding, and heat treatment. Commonly used soft magnetic powder materials include Fe–Si [8, 9], Fe–Si–Al [10, 11], Fe–Si–Cr [12, 13], carbonyl iron powder (CIP) [14], and Fe‐based amorphous/nanocrystalline alloys [15, 16]. Among them, Fe‐based amorphous/nanocrystalline soft magnetic powder has emerged as the most promising new soft magnetic material owing to its outstanding comprehensive soft magnetic properties [17, 18, 19, 20]. Surface coating treatment effectively enhances the resistivity of the powder material, improves surface adhesion, and ensures excellent formability [10, 21]. Compression molding is a critical process in the preparation of powder cores. The porosity, molding density, and particle uniformity during molding directly influence the soft magnetic properties of the final product [22, 23].

Applying high pressure to magnetic powder can yield high‐density powder core [24]. However, the high hardness and brittleness of Fe‐based amorphous/nanocrystalline powder limits the ability to achieve high‐density powder cores via traditional cold compaction [25]. Excessive forming pressure not only compromises the sphericity of amorphous/nanocrystalline particles, leading to increased core loss, but also exacerbates die wear, thereby hindering their large‐scale industrial application [26, 27]. Another challenge lies in the significant residual internal stress within powder cores formed under high pressure, which severely deteriorates their soft magnetic properties. Generally, the adverse effects of residual stress can be mitigated through high‐temperature or prolonged annealing processes [28]. However, the long‐range disordered structure of amorphous/nanocrystalline alloys hinders their ability to attain superior soft magnetic properties via high‐temperature or prolonged annealing treatments [17, 29, 30]. Consequently, the development of a forming method specifically adapted for amorphous/nanocrystalline powder cores is critically imperative.

In the present study, a unique ultrasonic‐assisted densification method, integrating ultrasonic vibration pressing‐cold pressing (UVP‐CP) hybrid process to fabricate amorphous/nanocrystalline toroidal powder core samples. The approach integrates an ultrasonic vibration system into a hydraulic press, enabling ultrasonic pre‐compaction of magnetic particles under low uniaxial pressure, followed by high‐pressure cold compaction for densification. This study investigates the correlation between density, soft magnetic properties, and pore distribution in powder cores fabricated with different forming processes. Additionally, it compares the magnetic performance of powder cores prepared by methods of conventional cold pressing (CP), ultrasonic vibration pressing (UVP), and UVP‐CP. This study correlates the synergistic effect of ultrasonic low‐pressure pre‐compaction and high‐pressure cold compaction with the soft magnetic properties of powder cores, while proposing a novel strategy for fabricating high‐performance amorphous/nanocrystalline powder cores.

## 2 Experiment

### 2.1 Materials

In this experiment, three magnetic powders with different median particle sizes were used. The Fe_73_Si_11_B_11_C_3_Cr_2_ amorphous spherical powder had a median particle size (*D*_50_) of approximately 27.0 µm. The Fe_73.5_Si_13.5_B_9_Cu_1_Nb_3_ (FINEMET) nanocrystalline spherical powder exhibited a smaller median particle size of about 5.5 µm, while the iron carbonyl powder (CIP) had an even smaller median particle size of approximately 4.5 µm. The morphologies and particle size distributions of FeSiBCCr, FeSiBNbCu, and CIP powders are shown in Figure S1. All magnetic powders were supplied by TIZ Advanced Alloy Technology Co., The anhydrous ethanol, acetone, and petroleum jelly employed in the experiments were sourced from China National Pharmaceutical Reagent Co., Ltd., while the epoxy resin (W6‐C) was supplied by Sichuan Chenghua Adhesive Industry Co., Ltd. All materials were utilized directly in sample preparation without additional purification.

### 2.2 Preparation of Magnetic Powder Cores

The Fe_73_Si_11_B_11_C_3_Cr_2_ magnetic powder was coated with an epoxy resin insulating binder to enhance its formability. Epoxy resin (2 wt.%, relative to the powder mass) was dissolved in acetone under ultrasonication. The magnetic powder was then introduced into the resin–acetone solution and stirred until the solvent had completely evaporated. Subsequently, the coated powder was dried under vacuum at 60°C for 30 min, followed by grinding and sieving. The same insulating coating procedure and materials were applied to Fe_73.5_Si_13.5_B_9_Cu_1_Nb_3_ and CIP powders.

Toroidal magnetic powder cores with an inner diameter of 8 mm and an outer diameter of 13 mm were fabricated using UVP, CP, and UVP‐CP forming processes, respectively. It should be noted that the geometric ratio of the samples used in this study is 1.625. According to the IEC 60404–6:2018+A1:2021 standard, the ring specimen should have dimensions such that the ratio of the outer to inner diameter is no greater than 1.4, and preferably less than 1.25, to achieve a sufficiently homogeneous magnetization of the test specimen. Due to current experimental resource constraints, strict adherence to this standard ratio was not achieved in this instance. The large ratio may cause a slightly increase in the permeability. This is adopted in commercial applications, even though it doesn't meet the standard evaluation. This deviation is documented here to ensure transparency, and we will resolve this in future studies by employing standardized geometries. The ultrasonic forming system consists of an ultrasonic vibration output system and a pressure output system. The UVP‐CP process involves pre‐compaction under low uniaxial pressure with ultrasonic vibration, followed by secondary cold pressing at a higher pressure. To eliminate internal stresses generated during compaction, the magnetic powder cores were annealed at 480°C for 1 h. The annealed samples were subsequently characterized.

### 2.3 Characterization Methods

The sample density was characterized through calculation. The phases present in the samples before and after annealing were analyzed by X‐ray diffraction (XRD, Bruker D8 Advance). Thermal stability and phase transition temperatures were characterized using differential scanning calorimetry (DSC, NETZSCH 404 C) at a heating rate of 40 K/min. Particle size distribution of the powder was measured by laser particle size analysis (HELOS‐OASIS). Furthermore, particle configuration, morphology, and pore structure were investigated using scanning electron microscopy (SEM, FEI Quanta FEG 250) and micro‐computed tomography (micro‐CT, Xradia 610). The effective permeability (*µ*_e_) and quality factor (*Q*) of powder cores were measured using an impedance analyzer (Agilent 4294 A) with contact electrodes in a two‐terminal configuration from 1 kHz to 110 MHz, the effective permeability was calculated as follows: [31]

(1) $$\mu_{e}=\frac{Ll_{e}}{\mu_{0}N^{2}A_{e}}\;$$

where, *L* is the inductance, *l*_e_ is the effective magnetic circuit length, *N* is the number of copper wire turns, *A*_e_ is the effective cross‐sectional area, and *µ*_0_ is the permeability of vacuum (4π × 10^−7^ H/m). Core loss (*P*_c_) was evaluated across 100 kHz –1 MHz using an AC B‐H analyzer (Iwatsu SY‐8218). The resistivity of powder cores was measured by hall effect testing system (8404‐CRX‐6.5K).

## 3 Results and Discussion

### 3.1 Ultrasound Parameter Exploration for UVP‐CP Powder Cores

A compaction pressure of 20 MPa was applied during the UVP stage and 500 MPa during the CP stage. By systematically varying ultrasonic amplitude and duration, the optimal ultrasonic parameters were determined through comprehensive measurements of density, permeability (*µ*_e_, with the applied field of 0.1 A/m at the frequency of 1 MHz), and core loss in samples fabricated from Fe–Si–B–C–Cr magnetic powders.

**FIGURE 1:**
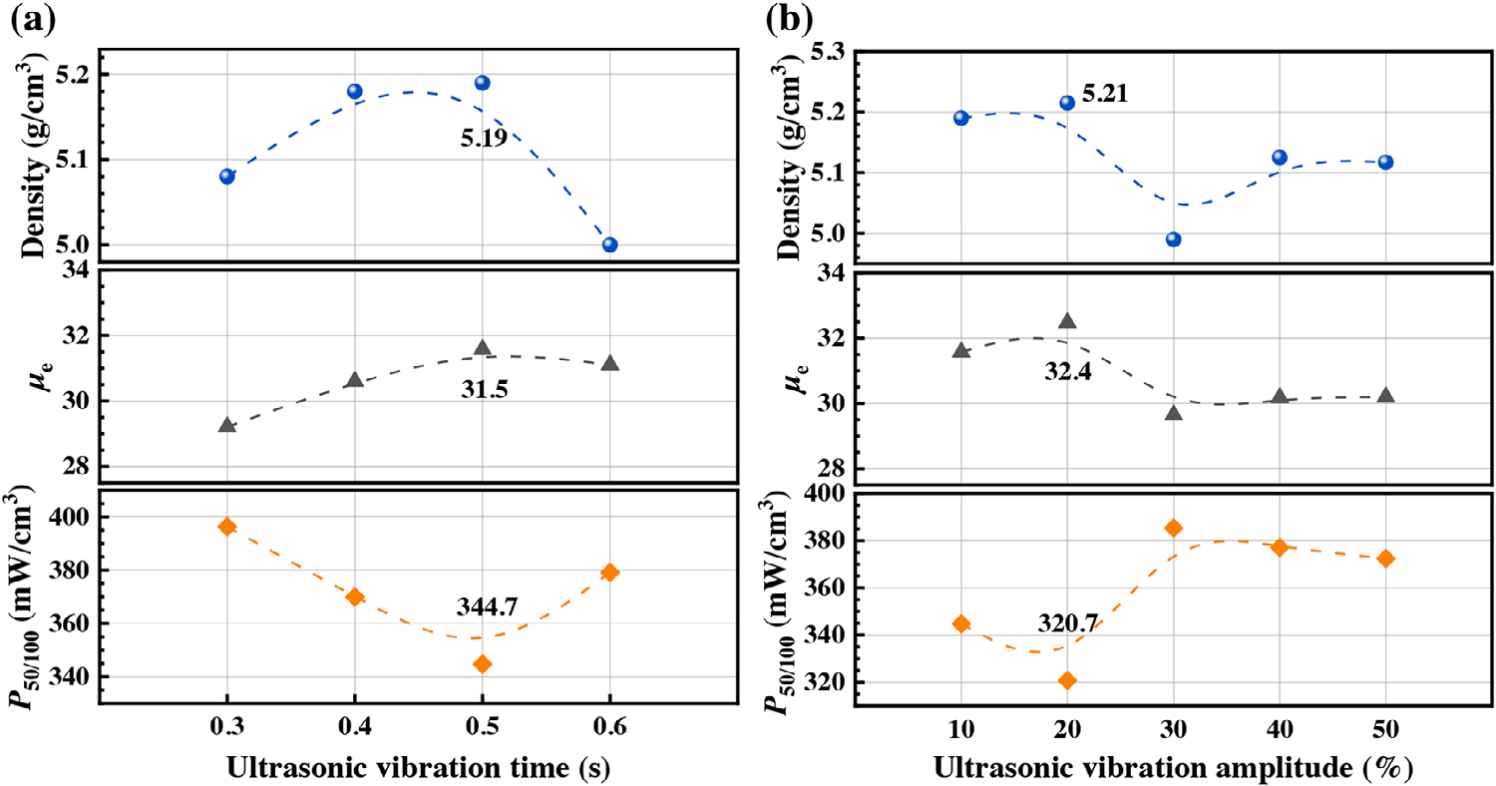
Effect of ultrasonic vibration (a) time and (b) amplitude on density, *µ*_e,_ and *P*_50mT/100 kHz_ of UVP‐CP powder cores.

The ultrasonic compaction system operated at 15 kHz. With the amplitude preset to 10%, the duration was varied from 0.3 to 0.6 s. As shown in Figure 1a, the density of UVP‐CP powder core initially increased then decreased with prolonged ultrasonic duration, peaking at 0.5 s, where the soft magnetic properties were also optimal. In Figure 1b, at 0.5 s ultrasonic duration, density similarly exhibited an initial rise followed by a decline with increasing amplitude, reaching maximum values at 20% amplitude, the soft magnetic properties also show the best under this condition. Consequently, the optimal UVP parameters were determined as 0.5 s ultrasonic duration and 20% ultrasonic amplitude, achieving a density of 5.19 ± 0.05 g/cm^3^, *µ*_e_ of 31.6, *P*_50mT/100 kHz_ of 320.7 mW/cm^3^.

### 3.2 Soft Magnetic Properties and Densification Mechanism of UVP‐CP Powder Cores

**FIGURE 2:**
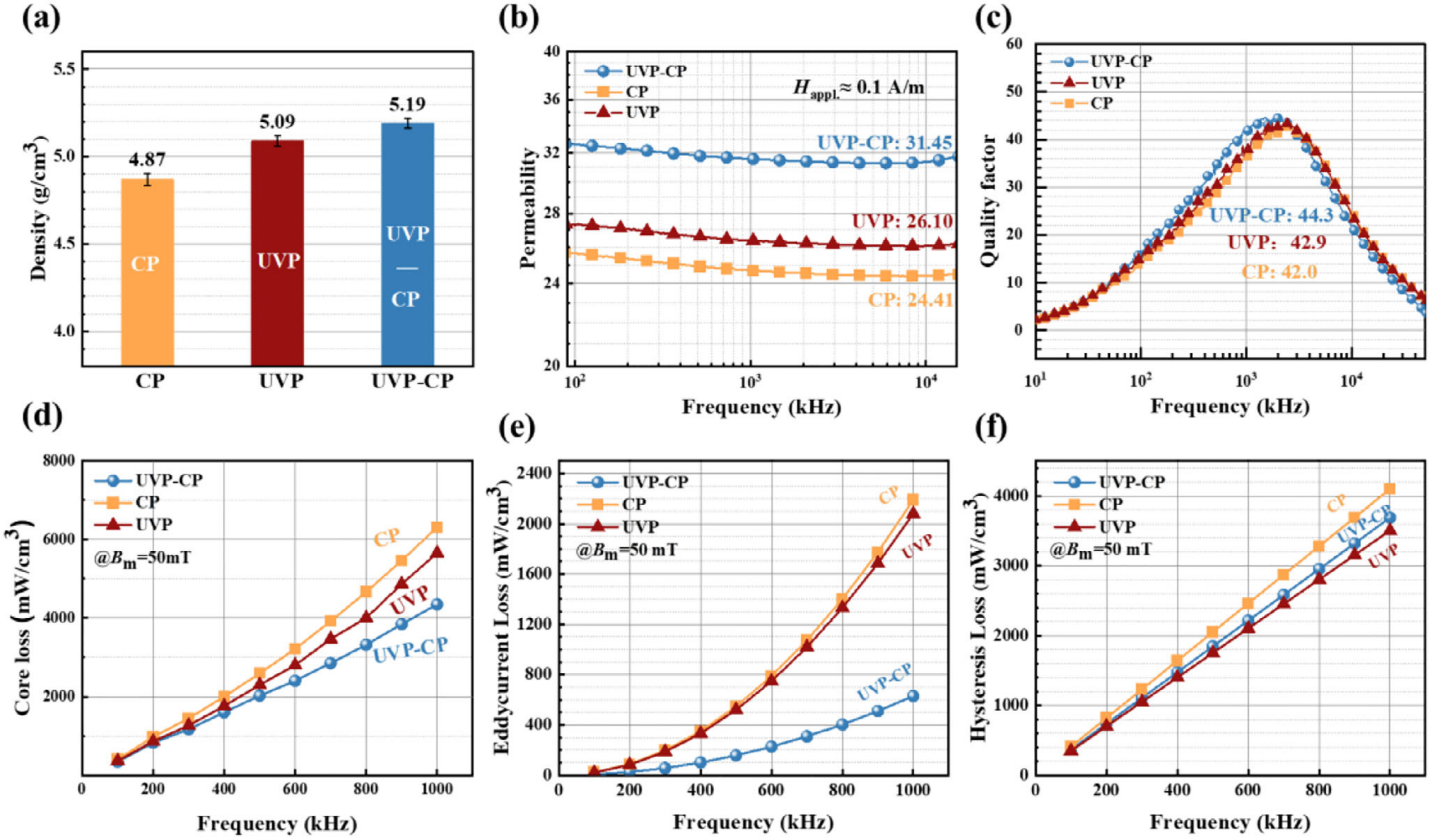
Comparisons in the density and magnetic properties of the powder cores samples: (a) density, (b) *µ*_e_, (c) *Q*, (d) *P*_50mT_, (e) *P*_e_, (f) *P*_h_.

To align with industrial integrated molded inductor fabrication [32, 33], cold pressing parameters for CP and UVP‐CP samples were set to 500 MPa, while ultrasonic pressure for UVP and UVP‐CP samples was 20 MPa, based on the pressure limits of the equipment. Figure 2a shows the density of the three samples. The UVP‐CP sample achieved the highest density of 5.19 g/cm^3^, whereas the UVP and CP samples reached lower values of 5.09 and 4.87 g/cm^3^, respectively. High density sample possess greater permeability, due to UVP‐CP sample has the maximum *µ*_e_ of 31.6, the *µ*_e_ of the UVP sample is 26, and the CP sample has the lowest *µ*_e_ of 24 (Figure 2b). The quality factor of the samples, as presented in Figure 2c, exhibits an inverse correlation with the core loss, where an increase in *Q* generally corresponds to a reduction in core loss. The UVP‐CP sample has the highest *Q* (= 44.3), the CP sample has the lowest *Q* (= 42). As shown in Figure 2d, core loss measurements conducted at 50 mT under different frequencies reveal that the UVP‐CP sample possesses the lowest core loss. The measured values are 320.7 mW/cm^3^ at 100 kHz and 4349.6 mW/cm^3^ at 1 MHz.

The total core loss *P*_c_ of soft magnetic powder cores generally consists of three components: hysteresis loss (*P*_h_), eddy current loss (*P*_e_), and residual loss (*P*_r_) [34]. At low frequencies, hysteresis loss constitutes the dominant portion of the total core loss and is strongly influenced by factors such as material density, internal stress, and magnetic anisotropy. As the frequency increases, eddy current loss gradually becomes the primary contributor to the total loss, which is mainly governed by the electrical resistivity, particle size, and related structural parameters. Notably, the residual loss is also observed in soft magnetic composite materials even at low and medium frequencies [35, 36, 37]. This component is closely associated with the magnetic domain dynamics and the non‐uniform magnetization processes occurring within the composite microstructure. Based on these frequency dependent characteristics, the three loss components can be expressed using the following equation:

(2) $$P_{c}=P_{h}+P_{e}+P_{r}=f\oint HdB+C_{e}B^{2}f^{2}d^{2}/\rho +C_{r}f^{1.5}B_{m}^{1.5}$$

where *f* is the frequency (kHz), *H* is the magnetic field strength (A/m), *B* is the magnetic flux density (T), *d* is the thickness of the material, *ρ* is the electrical resistivity, and *C*_e_ and *C*_r_ are material dependent constants. Hysteresis loss is proportional to both the frequency itself and the intrinsic magnetic properties, eddy current loss originates from resistive loss induced by alternating electric fields and is proportional to the square of the frequency. The variation curves of the separated *P*_e_ and *P*_h_, as derived from the above equation, are presented in Figure 2e,f, respectively. Among all samples, the UVP‐CP sample exhibits the lowest *P*_e_, with a value of 630 mW/cm^3^, representing a 70% reduction compared to that of the conventional CP sample (2190 mW/cm^3^). In addition, the hysteresis loss of both UVP and UVP‐CP is lower than that of CP powder core. The densities of UVP and UVP‐CP are very similar, and considering that in the UVP magnetic core, soft magnetic powders may be relatively concentrated, the hysteresis loss in the UVP core might be closer to that of the UVP‐CP core under low magnetic flux density and low frequency conditions. At the same time, the DC bias performance of the UVP‐CP powder core is clearly improved (Figure S2). The DC bias values (percent *µ*_e_) of the UVP‐CP powder core was greater than the CP powder core, especially at relatively high magnetic field intensities. Specifically, at 10000 A/m, the DC bias performance of the UVP‐CP powder core was maintained at 85.6%, which is significantly higher than that of the CP powder core (81.8%).

We also conducted a comparison to determine whether the performance differences between the UVP‐CP and CP processes are primarily due to the independent effect of ultrasonic vibration. The results, shown in Figure S3, indicate that the density and soft magnetic performance of the CP (20 MPa)‐CP samples are similar to those of the CP samples, but both are lower than the performance of the UVP‐CP samples. The significantly lower core loss of the UVP‐CP sample proves that the combined process is superior to using UVP or the CP method alone. This improvement is likely due to the ultrasonic vibration helps to arrange the particles more closely and uniformly before the final pressing, which results in a denser structure with less energy loss.

**FIGURE 3:**
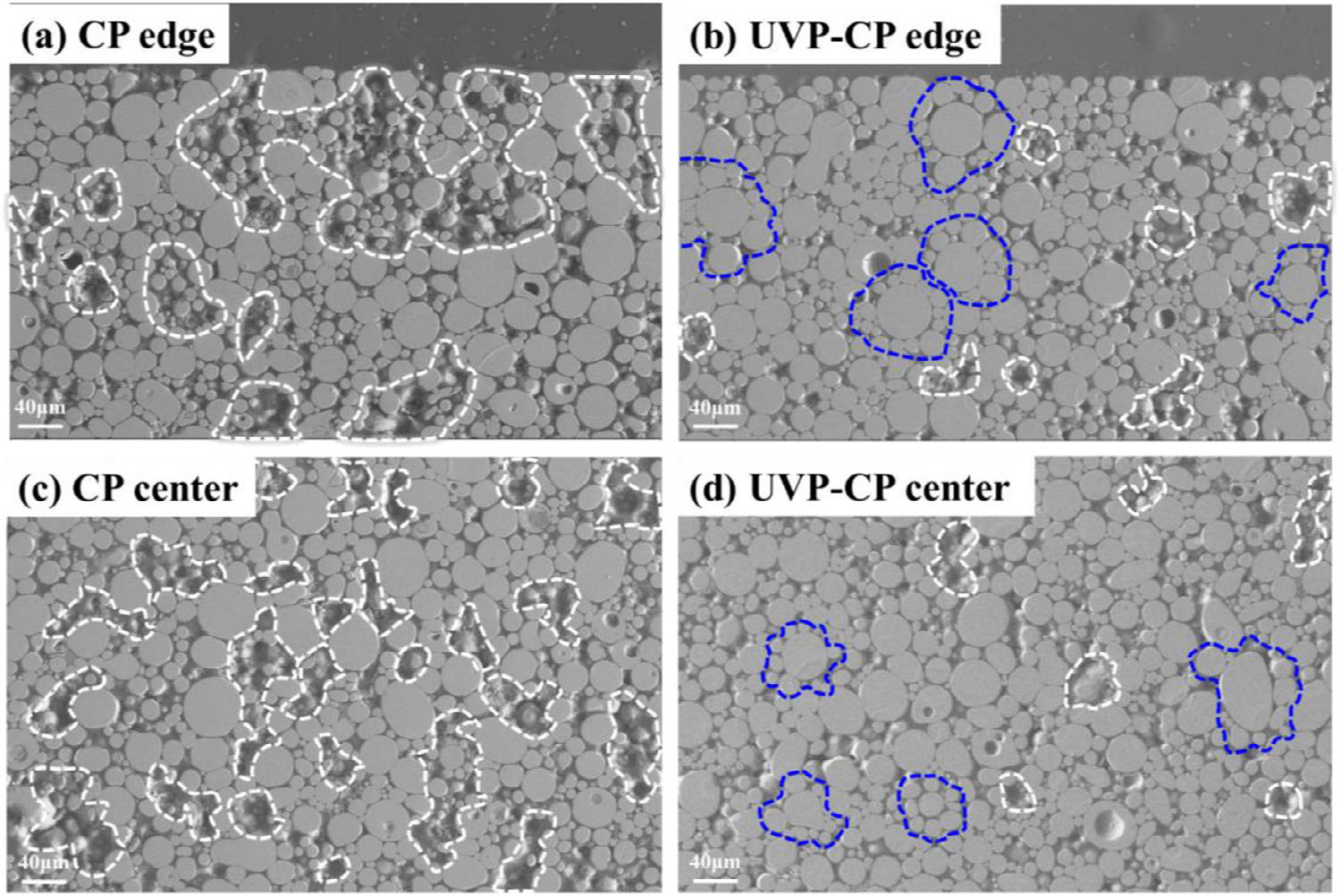
SEM morphologies of the edge parts in (a) CP core and (b) UVP‐CP core, the center parts in (c) CP core and (d) UVP‐CP core.

The soft magnetic properties of powder cores are closely correlated with the density and internal microstructure. A homogeneous structure, characterized by uniform distributions of magnetic particles and resin, enhances resistivity and minimizes eddy current core loss. Figure 3 presents SEM images comparing pore morphology at the edge and center of CP and UVP‐CP samples. The white dotted line marks the pores partially, and it is clear from Figure 3a,b showing that the pore area of the CP sample is much larger than UVP‐CP sample. In the central regions Figure 3c,d, a distinct pore contrast is observed: the CP sample display larger pore areas, while the UVP‐CP sample shows only minimal pores. This phenomenon arises because ultrasonic vibration during the UVP‐CP preloading stage reduces interparticle friction [38, 39], promoting uniform particle arrangement and thereby laying the groundwork for cold‐pressing densification. As shown in Figure 3b,d, the UVP‐CP sample exhibits a particle arrangement where interparticle voids among larger particles are filled with smaller particle (as blue dotted line marked), demonstrating uniform structures conducive to compaction. In contrast, the CP sample displays agglomerated small particles as shown in Figure 3a,c, which tends to form bridging structures under high pressure, impeding densification and potentially leading to particle fragmentation and degradation of soft magnetic properties. These microstructural observations confirm the superiority of the UVP‐CP process, which effectively minimizes pores and agglomeration to achieve superior magnetic performance. Furthermore, the morphology of magnetic powders prepared by different molding methods was shown in Figure S4. As observed in Figure S4a, a significant number of particles in the CP sample exhibit flattened surfaces due to uneven stress during the pressing process, leading to deformation. In contrast, the UVP sample (Figure S4b) maintains spherical particle morphology with no deformation, as the forming pressure in UVP is relatively low. The UVP‐CP sample (Figure S4c) shows a slight amount of particle deformation, but the number of deformed particles is significantly lower than that in the CP sample. This demonstrates that the UVP‐CP process promotes more uniform particle distribution, reducing the likelihood of particle deformation during compaction.

**FIGURE 4:**
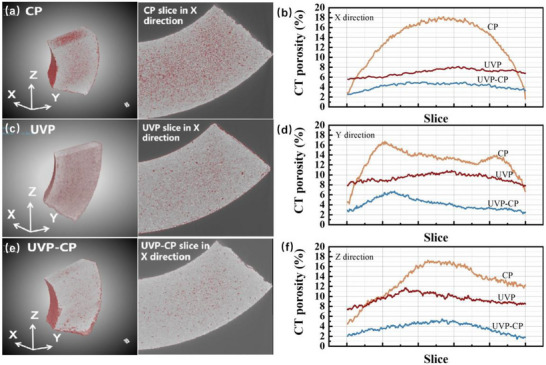
Micro‐CT analysis of pore structures: (a,c,e) 3D reconstruction images and (b,d,f) porosity distribution profiles for the CP, UVP, and UVP‐CP samples along the X, Y, Z directions, respectively.

Figure 4a,c,e illustrates the 3D pore distributions of CP, UVP, and UVP‐CP samples, with red regions representing pores. As shown in Figure 4a, the central area of the CP sample exhibits larger red regions compared to its edge area. This inhomogeneity arises from particle agglomeration during the cold‐pressing process. The process generates uneven stress and hinders effective densification, resulting in higher porosity and structural irregularity. Figure 4c displays the pore distribution of the UVP sample. Despite its relatively loose structure, the UVP sample exhibits smaller red pore regions than the CP sample, with pores uniformly distributed throughout the specimen, indicating homogeneous magnetic particle distribution. However, hindered densification occurred due to the lower forming pressure in the UVP process. Figure 4e presents the pore distribution of the UVP‐CP sample, which exhibits the smallest red pore regions. This improvement stems from ultrasonic‐induced friction reduction between particles, enabling uniform alignment of particles during preloading, followed by cold‐pressing densification to achieve a structurally homogeneous and dense bulk material [40, 41]. Figure 4b,d,f presents the pore percentage distributions along the X, Y, and Z directions for the CP, UVP, and UVP‐CP samples, respectively. It is clearly observable that the CP sample exhibits the highest porosity in all three orthogonal directions (X, Y, Z), while the UVP and UVP‐CP samples demonstrate much lower porosity. This indicates that ultrasonic treatment is an effective means of suppressing the formation of pores in powder cores. The UVP‐CP sample exhibits the lowest porosity, indicating that particles regulated by ultrasonic vibration can produce a powder core with both uniform particle orientation and a highly densified microstructure after subsequent cold pressing. At the same time, the CP sample demonstrates the largest porosity fluctuations, while UVP and UVP‐CP exhibit notably smaller variations. These findings confirm that ultrasonic effects critically govern particle alignment, thereby improving structural uniformity in powder cores.

### 3.3 Effect of Cold Pressing Pressure on the Properties of UVP‐CP Powder Cores

The effects of ultrasonic vibration and forming pressure on the densification mechanisms and soft magnetic properties were investigated by controlling the cold‐pressing pressure in the UVP‐CP process. For comparison, CP samples were also prepared under identical pressure conditions to elucidate the role of ultrasonic vibration. The density, permeability, quality factor, and core loss of samples from both methods were systematically evaluated.

**FIGURE 5:**
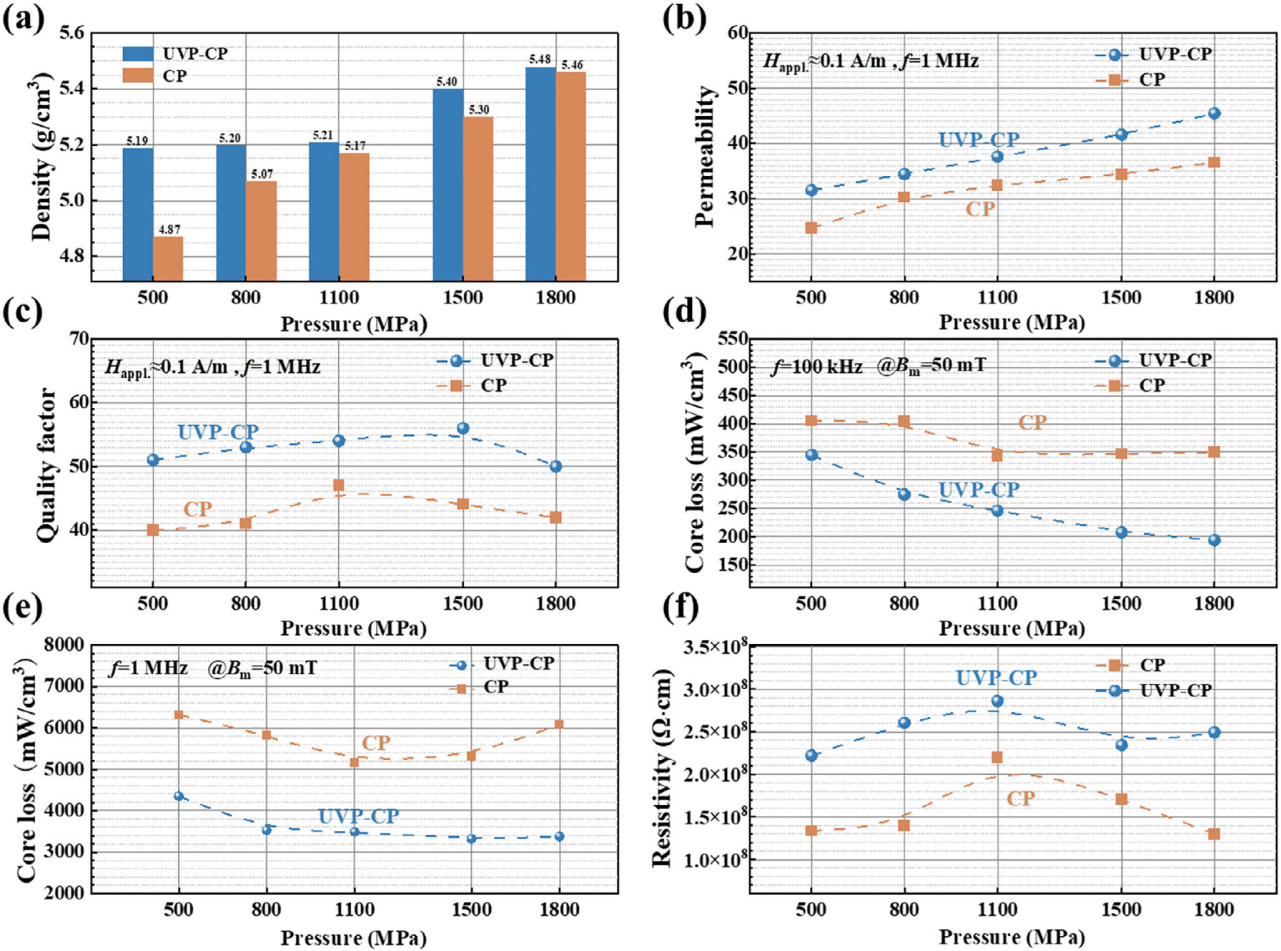
Comparisons in the pressure on (a) the density, (b) *µ*_e_ (at 0.1 A/m), (c) *Q*, (d) *P*_50mT/100 kHz_, (e) *P*_50mT/1 MHz_, (f) the resistivity.

As shown in Figure 5a, the density of both CP and UVP‐CP samples increase with increasing cold‐pressing parameters, while the density of UVP‐CP samples are consistently greater than those of CP samples across all pressure levels. Figure 5b reveals that UVP‐CP samples exhibit higher permeability than CP samples under all applied pressures. This enhancement originates from the preliminary ultrasonic vibration in the UVP‐CP process, which promotes particle rearrangement and establishes a homogeneous particle orientation structure (Figure 3b,d). The uniform particle arrangement within the powder cores effectively suppresses the formation of bridging/overlapping structures [19]. Figure 5c presents the quality factor (*Q*) of the samples, which is typically inversely correlated with core loss [42]. The *Q* of UVP‐CP samples is consistently superior to that of CP samples at all pressure levels. As depicted in Figure 5d, at low frequencies (100 kHz), the core loss of both CP and UVP‐CP samples follow a similar trend. With increasing pressure, the core loss initially decreases gradually for both sample types. However, once the pressing pressure exceeds 1100 MPa, the reduction in loss plateaus. Due to the ultrasound‐induced particle rearrangement, the more uniform structure of UVP‐CP samples results in lower loss compared to CP samples under all pressure conditions. From Figure 5e, it is evident that the high frequency core loss (1 MHz) of powder cores fabricated via the UVP‐CP method are lower than those produced by the CP method. For CP samples, as pressure increases, the high frequency core loss first decreases. However, when the forming pressure surpasses 1100 MPa, the loss subsequently increases. This is attributed to the sharp‐edged fractured particles piercing the insulating layer at pressures exceeding 1100 MPa, which reduces the sample resistivity and consequently leads to increased loss. In contrast, for UVP‐CP samples, the high frequency loss initially decreases and then tend to stabilize with increasing pressure. This behaviour stems from the uniform magnetic powder alignment achieved under ultrasonic vibration, which helps preserve the integrity of the insulating layers. This indicates that cores produced by the UVP‐CP method possess stable soft magnetic properties, making them more suitable for high frequency applications. Resistivity measurements shown in Figure 5f demonstrate that UVP‐CP samples possess higher resistivity compared to CP samples, which directly contributes to their reduced high frequency core loss.

The soft magnetic properties of powder cores fabricated under different pressure parameters using the three methods are summarized in Table 1. Analysis of the data in Table 1 indicates that the density, permeability, and loss of UVP‐CP samples at 500 MPa are comparable to those of CP samples at 1500 MPa. This suggests that incorporating ultrasound into the core forming process, leveraging its friction‐reducing effect to facilitate the formation of a uniform powder structure, enables the production of cores at lower pressures that achieve equivalent density and soft magnetic performance to those formed under significantly higher pressures. These comprehensive results unequivocally demonstrate that the UVP‐CP process consistently yields superior soft magnetic properties, including higher density, permeability, quality factor, and lower core loss across the entire range of pressing pressures when compared to the conventional CP method.

**TABLE 1:** The soft magnetic properties of FeSiBCCr powder core fabricated by CP, UVP, and UVP‐CP methods with different parameters.

Process	Pressure (MPa)	Density (g/cm^3^)	*µ*_e_	*P*_50mT/100 kHz_ (mW/cm^3^)	*P*_50mT/1 MHz_ (mW/cm^3^)
UVP‐CP	500	5.19	31.6	320.7	4349.6
	800	5.20	34.5	274.8	3526.6
	1100	5.21	37.6	246.0	3485.6
	1500	5.40	41.6	207.6	3317.4
	1800	5.48	45.5	194.0	3376.9
CP	500	4.87	24.7	405.8	6314.6
	800	5.07	30.2	404.3	5820.3
	1100	5.17	32.5	342.6	5166.8
	1500	5.30	34.4	346.9	5311.6
	1800	5.46	36.6	349.3	6083.6
UVP	20	5.09	26.4	519.2	5655.2

**FIGURE 6:**
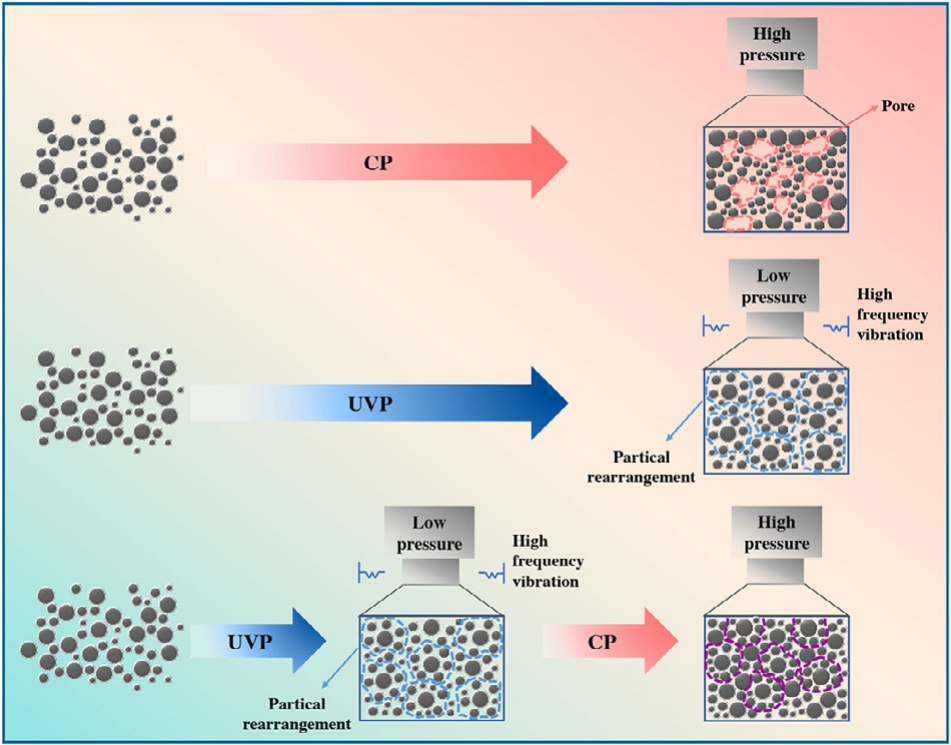
Schematic illustration of the CP, UVP, UVP‐CP process.

The superior soft magnetic properties and structural advantages observed in the UVP‐CP samples can be attributed to the fundamental differences in the microstructural evolution during the forming processes, as schematically illustrated in Figure 6. The conventional CP process has limited capability in particle rearrangement, often resulting in powder cores with high porosity and non‐uniform powder distribution, which hinders the achievement of high density. In contrast, the enhancement of particle rearrangement by ultrasonic vibration stems from its friction‐reducing effect during the initial forming stage. This effect promotes particle rearrangement and establishes a homogeneous particle orientation structure, which effectively suppresses the formation of bridging or overlapping structures and facilitates the production of high‐density samples. However, according to the mechanical equilibrium model, significant particle rearrangement induced by high frequency ultrasonic vibration may be restricted when the applied pressure becomes excessively high [38]. In this study, a lower pressure was therefore selected for the ultrasonic vibration stage to avoid imposing large external constraints on the powder compact during particle motion. Since such a low pressure is insufficient to achieve the required densification, a subsequent high pressure compaction step was introduced to obtain higher density. The ultrasonic treatment assists fine particles in entering interstitial regions, improving packing uniformity, while the secondary high pressure compaction ensures adequate densification. This combination of improved structural uniformity and increased density contributes to reduced magnetic hysteresis, enhanced permeability, and lower core loss.

### 3.4 Based on Gradation and UVP‐CP Method to Fabricate High‐Permeability and Low‐Loss Powder Core

Although amorphous and nanocrystalline magnetic powders exhibit excellent soft magnetic properties, their inherently high hardness limits their ability to undergo sufficient deformation during compaction, thereby restricting densification and potentially affecting the final soft magnetic performance. Composite grading of magnetic powders with varying sizes and chemical compositions not only effectively fills pores within the powder core but also synergizes the performance of different kind of powders, surpassing the soft magnetic properties limits of single‐component powders to achieve superior comprehensive soft magnetic performance [14, 43, 44]. In this part of the work, amorphous powders (Fe–Si–B–C–Cr) are employed as the base material. Considering that industrial magnetic powder cores often require particle‐size grading or functional enhancement through the incorporation of additional powders, we introduce two representative commercial magnetic materials Fe–Si–B–Nb–Cu (FINEMET) and carbonyl iron powder (CIP) into the amorphous matrix. By integrating composite grading strategies, we systematically compare different forming methods to provide an initial assessment of the UVP‐CP process in a multi‐component powder system that more closely reflects practical applications.

**FIGURE 7:**
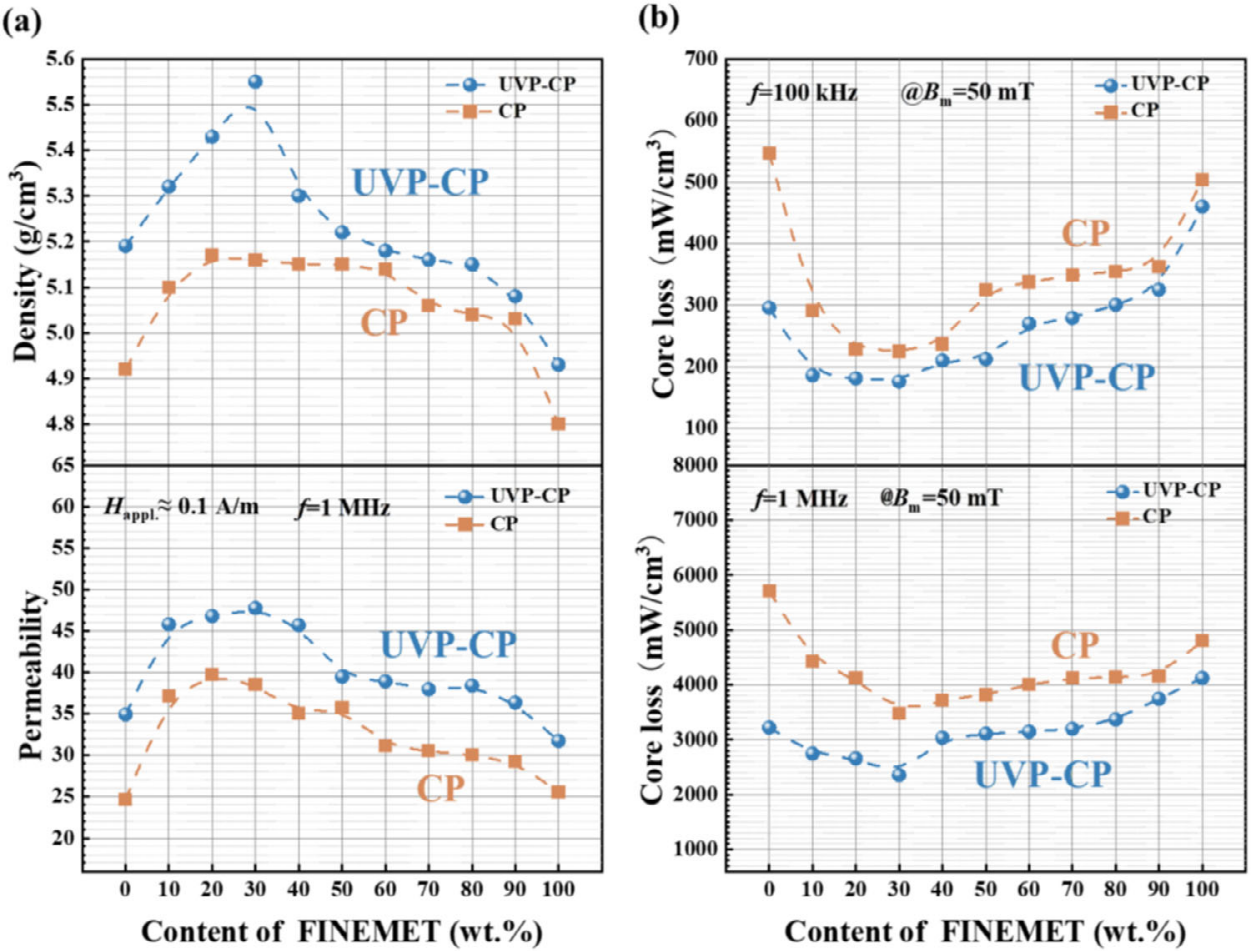
Comparison in soft magnetic properties of FINEMET powder cores prepared by CP and UVP‐CP processes: (a) the density and *µ*_e_, (b) *P*_50mT/100 kHz_ and *P*_50mT/1 MHz_.

As shown in the upper picture of Figure 7a, the density of FINEMET composite powder cores increases significantly with the FINEMET content in the range of 10%–30%, reaching a maximum of 5.55 g/cm^3^ for UVP‐CP samples at 30% FINEMET content (see also Table 2), and the bottom picture of Figure 7a shows the *µ*_e_ has the same tend. However, when FINEMET content exceeds 30%, the density gradually decreases. This phenomenon is attributed to the pore‐filling effect of small FINEMET particles at an optimal concentration, which reduces porosity significantly. However, overloading with fine particles triggers agglomeration phenomena, which generate enlarged interstitial voids and degrade the density. As shown in Figure 7b, the core loss was measured at low frequency and high frequency are shown in the upper and bottom image, respectively. It is evident from Figure 7b that the CP sample exhibits higher loss than the UVP‐CP sample at both high and low frequencies. This can be quantitatively verified in Table 2, where the core loss (*P*_50mT/100 kHz_ and *P*_50mT/1 MHz_) are systematically listed for all compositions and processes. The higher density of the UVP‐CP samples results in lower hysteresis loss, while the more uniform powder alignment and reduced particle fracture in UVP‐CP samples also help minimize resin cracking on particle surfaces, thereby reducing eddy current loss in the core. In summary, based on the comprehensive data presented in Table 2, the UVP‐CP prepared composite powder core with 30% FINEMET exhibits optimal soft magnetic properties: the highest *µ*_e_ of 47.8 and the lowest core loss with *P*_50mT/100 kHz_ of 175.8 mW/cm^3^ and *P*_50mT/1 MHz_ of 2353.4 mW/cm^3^.

**TABLE 2:** The soft magnetic properties of FINEMET composite powder cores fabricated by UVP‐CP and CP.

Materials	Process	Density (g/cm^3^)	*µ*_e_	*P*_50mT/100 kHz_ (mW/cm^3^)	*P*_50mT/1 MHz_ (mW/cm^3^)
FeSiBCCr +10%FINEMET	UVP‐CP	5.32	45.8	185.7	2746.8
	CP	5.10	37.1	291.3	4425.9
FeSiBCCr +20%FINEMET	UVP‐CP	5.43	46.8	181.1	2658.4
	CP	5.17	39.8	227.6	4120.2
FeSiBCCr +30%FINEMET	UVP‐CP	5.55	47.8	175.8	2353.4
	CP	5.16	38.6	224.9	3477.8
FeSiBCCr +40%FINEMET	UVP‐CP	5.30	45.7	210.1	3033.2
	CP	5.15	35.0	237.0	3721.5
FeSiBCCr +50%FINEMET	UVP‐CP	5.22	39.5	212.1	3106.1
	CP	5.15	35.8	325.1	3813.7
FeSiBCCr +60%FINEMET	UVP‐CP	5.18	39.0	269.8	3144.1
	CP	5.14	31.1	337.6	4006.7
FeSiBCCr +70%FINEMET	UVP‐CP	5.16	38.0	279.1	3193.2
	CP	5.06	30.5	346.2	4118.9
FeSiBCCr +80%FINEMET	UVP‐CP	5.15	38.4	300.5	3364.1
	CP	5.04	30.0	355.0	4138.3
FeSiBCCr +90%FINEMET	UVP‐CP	5.05	36.4	325.1	3743.9
	CP	5.03	29.2	363.1	4157
FINEMET	UVP‐CP	4.93	31.8	460.1	4122.5
	CP	4.93	5.6	503.9	4798.2

Micro‐CT images and porosity of CP and UVP‐CP samples with 30% FINEMET are compared in Figure S5. The CP sample exhibits extensive red‐marked pores formed by aggregated fine FINEMET particles, whereas UVP‐CP samples show no such large‐scale porosity. This confirms that UVP‐CP processing disrupts particle agglomeration and optimizes particle packing through size regulated alignment. Furthermore, powder cores demonstrate superior graded particle filling compared to single‐component systems, as evidenced by their lower porosity variation. UVP‐CP samples exhibit even further reduced porosity variation. These results collectively verify that UVP‐CP cores achieve more uniform and compact microstructures than conventional CP‐processed counterparts.

**FIGURE 8:**
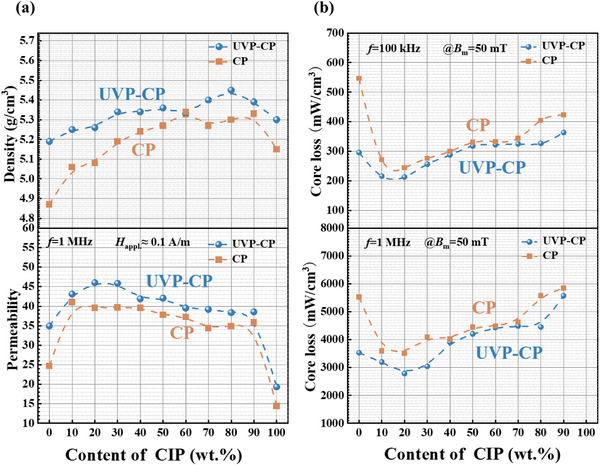
The comparisons of the soft magnetic properties of CIP composite cores fabricated by UVP‐CP and CP methods. (a) the density and *µ*_e_, (b) *P*_50mT/100 kHz_, *P*_50mT/1 MHz_.

Figure 8a demonstrates that UVP‐CP processed powder cores which exhibiting higher density and *µ*_e_ compared to CP counterparts. As detailed in Table 3, the soft magnetic properties of FeSiBCCr‐CIP composite cores prepared by both UVP‐CP and CP methods confirm this trend: UVP‐CP samples consistently achieve higher density and *µ*_e_ values across all CIP content levels. However, CIP containing composite powder cores exhibit slightly higher loss than FINEMET‐based composites, which can be attributed to processing induced effects such as particle deformation, internal stress accumulation, and microstructural nonuniformity during compaction. This can be observed in the measured core loss data in Table 3, where the loss values of CIP composites are generally higher than those of FINEMET composites (Table 2) under comparable content and processing conditions. Optimal soft magnetic performance is achieved at 20% CIP content, yielding *µ*_e_ of 46 with *P*_50mT/100 kHz_ of 256.46 mW/cm^3^ and *P*_50mT/1 MHz_ of 3042.4 mW/cm^3^, respectively.

**TABLE 3:** The soft magnetic properties of CIP composite powder cores fabricated by UVP‐CP and CP.

Materials	Process	Density (g/cm^3^)	*µ*_e_	*P*_50mT/100 kHz_ (mW/cm^3^)	*P*_50mT/1 MHz_ (mW/cm^3^)
FeSiBCCr +10%CIP	UVP‐CP	5.25	43.1	215.76	3194.1
	CP	5.06	41.0	271.2	3589.5
FeSiBCCr +20%CIP	UVP‐CP	5.26	46.0	213.06	2784.3
	CP	5.08	39.6	244.26	3509.8
FeSiBCCr +30%CIP	UVP‐CP	5.34	45.9	256.46	3042.4
	CP	5.19	39.7	275.73	4093.4
FeSiBCCr +40%CIP	UVP‐CP	5.34	41.9	287.16	3901.0
	CP	5.24	39.5	299.44	4020.2
FeSiBCCr +50%CIP	UVP‐CP	5.36	42.1	317.6	4202.5
	CP	5.27	37.8	329.75	4445.4
FeSiBCCr +60%CIP	UVP‐CP	5.33	39.5	322.15	4431.8
	CP	5.34	37.2	332.76	4489.2
FeSiBCCr +70%CIP	UVP‐CP	5.40	39.2	324.85	4487.7
	CP	5.27	34.3	343.7	4634.6
FeSiBCCr +80%CIP	UVP‐CP	5.45	38.4	326.72	4451.7
	CP	5.30	34.9	404.17	5578.8
FeSiBCCr +90%CIP	UVP‐CP	5.39	38.6	363.36	5574.5
	CP	5.33	35.8	423.51	5846.1
CIP	UVP‐CP	5.30	19.3	4311.6	—
	CP	5.15	14.4	9418.2	—

Table 4 lists the core loss, permeability, density, and molding pressure achieved in this work, along with the corresponding properties from relevant previous studies. As demonstrated by ref. [22], ref. [23], and the present work, the application of ultrasound enables the fabrication of high density magnetic cores under relatively low molding pressure, which significantly improves core loss and is beneficial for enhancing both permeability and DC bias capability. Compared with the commonly used CP method for preparing soft magnetic powder cores, these approaches exhibit notable advantages.

**TABLE 4:** Comparison of core loss, *µ*_e_, density, and molding pressure with reported soft magnetic powder cores in the literature.

Core loss (mW•cm^−3^)	*µ*_e_	Density (g•cm^−3^)	Pressure (MPa)	Refs.
*P*_50mT/100 kHz_ = 175.8	47.8	5.55	500	This work
*P*_50mT/100 kHz_ = 220	30	5.10	20	[22]
*P*_100mT/100 kHz_ = 13.73	43.3	—	6.2	[23]
*P*_50mT/1 MHz_ = 2290	48.2	5.51	1800	[45]
*P*_50mT/100 kHz_ = 47	60	6.02	1800	[46]
*P*_50mT/100 kHz_ = 312.5	39.1	—	1500	[47]
*P*_50mT/100 kHz_ = 222	46.5	5.10	1150	[48]
*P*_50mT/500 kHz_ = 640	44.6	6.4‐6.6	1500	[49]
*P*_50mT/500 kHz_ = 149	68	—	1600	[50]
*P*_50mT/500 kHz_ = 154.4	95.3	6.04	160	[51]
*P*_50mT/50 kHz_ = 590	69	—	1000	[52]
*P*_20mT/1 MHz_ = 684.96	30.8	—	1000	[53]
*P*_50mT/1 MHz_ = 2625	20	5.46	1000	[54]
*P*_50mT/300 kHz_ = 614	38.2	5.80	2050	[31]
*P*_50mT/100 kHz_ = 265	67	—	1800	[55]
*P*_10mT/2.5 MHz_ = 248.48	24	5.4‐5.5	600	[56]

## 4 Conclusion

This study systematically optimized the ultrasonic vibration parameters for amorphous/nanocrystalline powder cores and evaluated the soft magnetic properties of three compaction techniques: CP, UVP, and UVP‐CP. The UVP‐CP process enabled the production of powder cores under a low pressure of 500 MPa, exhibiting excellent soft magnetic properties comparable to those of CP samples prepared at 1800 MPa. A uniform pore structure combined with low porosity was identified as a critical factor for achieving optima soft magnetic performance. Furthermore, by incorporating ultrafine nanocrystalline powders and applying the UVP‐CP method, the resulting composite powder cores reached a density of 5.55 g/cm^3^, an effective permeability of 47.8, and a core loss of 175.8 mW/ cm^3^ at 100 kHz and 2353.4 mW/ cm^3^ at 1 MHz under 50 mT. These findings demonstrate that the UVP‐CP method is highly suitable for fabricating high‐performance soft magnetic composites for integrated inductors.

## Conflicts of Interest

The authors declare no conflicts of interest.

## Supporting information




**Supporting File**: advs74098‐sup‐0001‐SuppMat.docx.

## Data Availability

The data that support the findings of this study are available from the corresponding author upon reasonable request.
