# Supplementary material for: Designing Advanced Soft Magnetic Powder Cores with Ultralow Energy Loss by Combining Ultrasonic and Static Compaction
Source: Adv Sci (Weinh). 2026 Jan 26;13(19):e22847. doi: 10.1002/advs.202522847 (PMC13045464; doi:10.1002/advs.202522847)
Supplement: Supplementary file 1 — Supporting File: advs74098‐sup‐0001‐SuppMat.docx. [file ADVS-13-e22847-s001.docx]

Supplementary Material

**Designing advanced soft magnetic powder cores with ultralow energy loss by combining ultrasonic and static compaction**

Xiaoying Huang^a,b,1^, Dongming Zhu^a,1^, Xiao Jin^a^, Yanan Chen^a,*^, Meng Gao^a,c^, Xuanyuan Zhang^a,d^, Min Nie^e^, Bingnan Yao^a^, Wei Xu^a^, Wenbo Wang^f^, Xiaojun Zhao^d^, Juntao Huo^a^, Mingliang Xiang^a,*^, Jun-Qiang Wang^a,c,*^, Yan Zhang^a,c,*^

^a^ *Ningbo Institute of Materials Technology and Engineering, Chinese Academy of Science, Zhejiang Key Laboratory of Magnetic Materials and Applications, Ningbo 315201, China*

^b^ *School of Materials Science and Chemical Engineering, Ningbo University, Ningbo 315211, China*

^c^ *Center of Materials Science and Optoelectronics Engineering, University of Chinese Academy of Sciences, Beijing 100049, China*

^d^ *Department of Electrical Engineering, North China Electric Power University, Baoding 071003, China*

^e^ *Shenzhen Sunlord Electronics Co., Ltd., Shenzhen 518110, China*

^f^ *North China Electric Power University, No. 2 Beinong Road, Beijing ,102206, China*

^*^ Corresponding authors.

E-mail addresses: yzhang@nimte.ac.cn, chenyanan@nimte.ac.cn, xiangmingliang@nimte.ac.cn, and jqwang@nimte.ac.cn

^1^ These authors contributed equally to this work.

The supplementary material includes: Figure S1-S5.


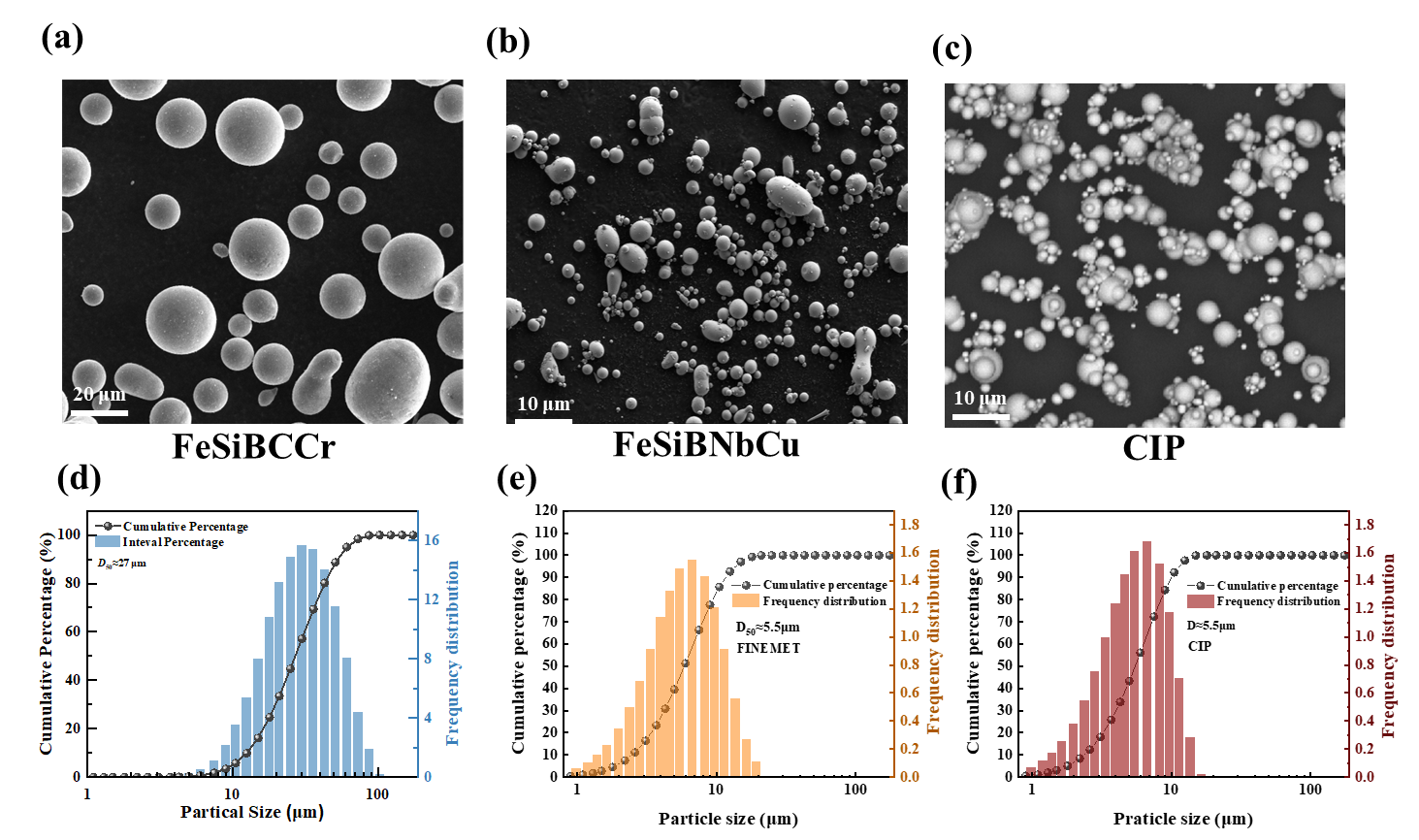


Figure S1. (a-c) SEM micrographs of Fe-Si-B-C-Cr, Fe-Si-B-Nb-Cu and carbide iron powder (CIP), respectively. (d-f) Laser particle size distribution of Fe-Si-B-C-Cr, Fe-Si-B-Nb-Cu and CIP powders.

Figure S2. DC bias performance of the UVP-CP, CP and UVP powder cores.

Figure S3. Comparisons in the density and magnetic properties of the UVP, CP, UVP (20 MPa)-CP and CP (20 MPa)-CP powder cores samples: (a) density, (b) *μ*e, (c) *P*_50mT_, (d) *Q*

Figure S4. SEM images of magnetic powder cores prepared by different molding methods: (a) CP, (b) UVP, and (c) UVP-CP.


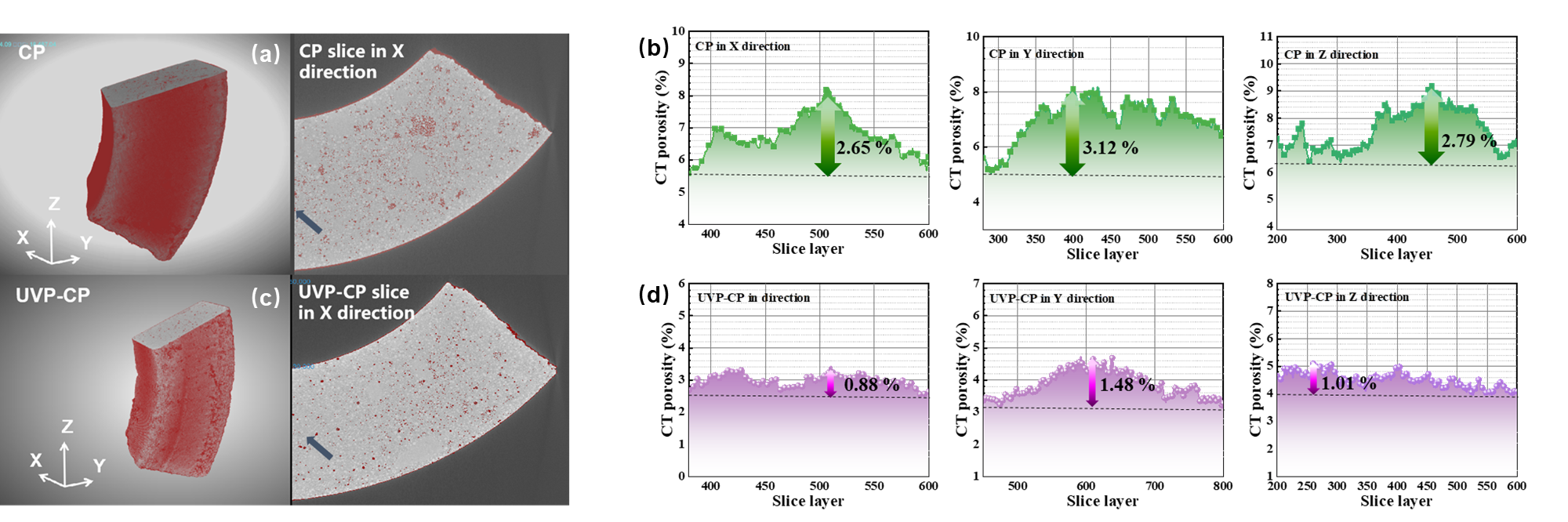


Figure S5. (a) Micro-CT scanning image and (b) micro-CT slice porosity analysis of CP sample with 30% FINEMET powder, (c) Micro-CT scanning image and (d) micro-CT slice porosity analysis of UVP-CP sample with 30% FINEMET powder.
